# Cancer Spheroids Embedded in Tissue-Engineered Skin Substitutes: A New Method to Study Tumorigenicity In Vivo

**DOI:** 10.3390/ijms25031513

**Published:** 2024-01-26

**Authors:** Martin A. Barbier, Karel Ferland, Henri De Koninck, Emilie J. Doucet, Ludivine Dubourget, MinJoon Kim, Bettina Cattier, Amélie Morissette, Mbarka Bchetnia, Danielle Larouche, Dong Hyun Kim, Guillaume St-Jean, Lucie Germain

**Affiliations:** 1 The Tissue Engineering Laboratory (LOEX), Université Laval’s Research Center, Quebec, QC G1V 0A6, Canada; martin-alexandre.barbier.1@ulaval.ca (M.A.B.); karel.ferland.1@ulaval.ca (K.F.); henri.de-koninck.1@ulaval.ca (H.D.K.); emilie.doucet.4@ulaval.ca (E.J.D.); ludivine.dubourget.1@ulaval.ca (L.D.); mjk042821@gmail.com (M.K.); bettina.cattier.1@ulaval.ca (B.C.); amelie.morissette@crchudequebec.ulaval.ca (A.M.); mbarka.bchetnia@crchudequebec.ulaval.ca (M.B.); danielle.larouche@crchudequebec.ulaval.ca (D.L.); terios92@hanmail.net (D.H.K.); 2 Department of Surgery, Faculty of Medicine, Université Laval, Quebec, QC G1V 0A6, Canada; 3 Regenerative Medicine Division, CHU de Québec-Université Laval Research Centre, Quebec, QC G1J 1Z4, Canada; 4 Department of Dermatology, CHA Bundang Medical Center, CHA University, 59 Yatap-ro, Bundang-gu, Seongnam-si 463-712, Gyeonggi-do, Republic of Korea; 5 Department of Pathology and Microbiology, Faculty of Veterinary Medicine, Université de Montréal, Saint-Hyacinthe, QC J2S 2M2, Canada; guillaume.st-jean.2@umontreal.ca

**Keywords:** cancer spheroids, tissue engineering, skin, cell culture, tissue-engineered substitutes, grafting, tumor

## Abstract

Tumorigenic assays are used during a clinical translation to detect the transformation potential of cell-based therapies. One of these in vivo assays is based on the separate injection of each cell type to be used in the clinical trial. However, the injection method requires many animals and several months to obtain useful results. In previous studies, we showed the potential of tissue-engineered skin substitutes (TESs) as a model for normal skin in which cancer cells can be included in vitro. Herein, we showed a new method to study tumorigenicity, using cancer spheroids that were embedded in TESs (cTES) and grafted onto athymic mice, and compared it with the commonly used cell injection assay. Tumors developed in both models, cancer cell injection and cTES grafting, but metastases were not detected at the time of sacrifice. Interestingly, the rate of tumor development was faster in cTESs than with the injection method. In conclusion, grafting TESs is a sensitive method to detect tumor cell growth with and could be developed as an alternative test for tumorigenicity.

## 1. Introduction

In vivo tumorigenic assays are widely used to evaluate the safety of cell-based therapies [1,2,3]. These assays usually rely on the ability of cells to develop local tumors when injected in an animal. A cell-based therapy will be considered non-tumorigenic, and therefore safe, if the injected cells used in the treatment do not develop tumors. According to the U.S. Food and Drugs Administration (FDA), the choice of the animal model, the definition of positive controls, the duration of the assay and the selection of controls need to be considered before performing a tumorigenicity test [4]. A commonly employed assay is the subcutaneous injection of cells in nude (nu/nu) mice [5]. It consists of inoculating each animal with 10 × 10^6^ of the cells proposed for the therapy, as well as tumorigenic cells, such as HeLa, melanoma or other cancer cells, as a positive control [4,6,7,8]. At least 10 animals per group (test and control) must be inoculated, and 9 out of 10 control animals must develop a tumor for the test to be valid [4].

The FDA also indicates that cells with a low tumorigenic potential can require up to 7 months to develop tumors [4]. Furthermore, for more complex cell-based therapies, each cell type needs to be tested separately, therefore increasing the number of animals required. Consequently, tumorigenic assays are time-consuming, costly and make use of many animals to be considered valid, raising ethical concerns. Furthermore, longer tests can increase the risk of spontaneous tumor formation, thus augmenting the false positive rate [4]. The co-inoculation of cell lines with basement membrane extracts such as Matrigel and Culturex is often employed to enhance tumor growth and the prevalence of metastasis [9,10,11,12]. Indeed, when co-inoculated with Matrigel, animals injected with breast cancer cell lines develop tumors faster than the ones injected without, effectively reducing the duration of the assay [9]. However, Matrigel does not mimic the physiological environment of the cells and may contribute to the development of tumors even when combined with non-tumorigenic cells, once again possibly creating false positive results. A previous study has demonstrated that non-tumorigenic NIH-3T3 cells can form local, vascularized and invasive tumors when co-inoculated with Matrigel [13], but the experiment has yet to be conducted with human cells. All in all, tumorigenic assays can be difficult to perform when the number of cells is low, as well as time and resource consuming, which encourages the development of alternative methods to test tumorigenicity for new and specific cell- or tissue-based therapies. 

Tissue-engineered skin substitutes (TESs) are bilayered skin constructs made with fibroblasts and keratinocytes. They exploit the capability of cells to form an organized three-dimensional tissue without using any exogenous scaffold or biomaterial. The result is a skin substitute that shares many properties with native human skin that can be produced from autologous cells and transplanted to treat full-thickness skin injuries of severely burned patients [14]. TESs made with human cells have been well studied and are known to replicate the skin microenvironment in vitro and in vivo when grafted onto athymic mice [15]. TESs produced with cells from healthy individuals do not form tumors. However, these substitutes, when produced with cancer-derived cells such as basal cell carcinoma (BCC), mimic the characteristics of BCC in vitro by showing basaloidal cells surrounded by a fibromyxoid stroma and the abnormal localization of keratin 10 and keratin 15 [16]. Additionally, the inoculation of melanoma spheroids in TESs also causes the development of melanocytic lesions that are characterized by proliferating melanoma cells which invade the dermo–epidermal junction [17,18]. Thus, the permissive environment of the TES model for the development of tumors makes it a great tool to study tumorigenicity in vitro. 

Nevertheless, in vitro experiments showed that the inoculation of tumor cells in TESs produces relatively small tumors that are not macroscopically perceptible [17]. Consequently, a histological analysis or an abnormal marker expression search must be performed to detect the presence of a tumor using this model. To extend the TES maturation period and allow long-term studies, TESs can be grafted onto athymic mice, since both fibroblasts and keratinocytes revert to their homeostatic behavior once grafted. This grafting procedure also allows preclinical assays of the product to assess the efficiency of skin substitutes for up to 6 to 12 months [15,19]. We have previously shown that no abnormal features were observed in TESs produced using healthy human donor cells and grafted on athymic mice for extended periods of time [15]. 

In the present study, we hypothesized that cancer cells will form a tumor of noticeable size within 3 to 6 months if they were present in the grafted TESs. We believe that such a test could be an effective way of assessing tumorigenicity, while better mimicking the skin microenvironment. We evaluated the potential of cancer cell spheroids embedded in TESs (cTESs) to develop a tumor following engraftment on athymic mice and contrasted this approach with the subcutaneous cell injection method. Our aim was to evaluate an alternative to the aforementioned tumorigenicity assay for tissue-engineered substitutes to reduce costs, animal use and time in future preclinical studies.

## 2. Results

### 2.1. Overview of the Subcutaneous Injection Method Compared to the Grafting of Tissue-Engineered Skin (TES) and Cancer-Cell-Spheroid-Containing Tissue-Engineered Skin (cTES)

**Figure 1:**
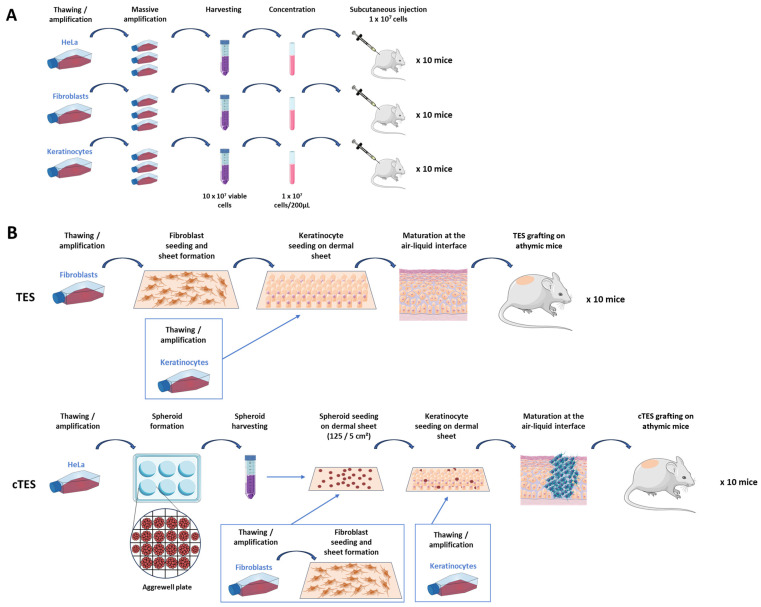
Schematic Representation of the Two Tumorigenic Assays Tested. (**A**) Schematic representation of the subcutaneous injection and (**B**) healthy tissue-engineered skin substitute (TES) and cancerous tissue-engineered skin substitute (cTES) method.

Two methods were compared for the evaluation of the tumorigenic potential. The first test involved the subcutaneous injection of cells to nude mice. It required a massive cell expansion in culture to reach at least 100 million viable cells for each cell type of interest, fibroblasts and keratinocytes in the case of skin substitutes, and cancer cells such as HeLa cells, to inject 10 animals with 10 million cells each (Figure 1A). The second method tested was the grafting of tissue-engineered skin substitutes produced with fibroblasts and keratinocytes either with cancer spheroids (cTES) or without (TES). To produce the skin substitute, dermal sheets were made using the self-assembly approach, which relies on a culture medium supplemented with ascorbic acid to promote the assembly of the extracellular matrix. HeLa cells were amplified and cultured in Aggrewell plates to form spheroids that were seeded on a dermal sheet. Keratinocytes were then added over the spheroids and the dermal sheet. Finally, after two more dermal sheets were stacked under the tissue-engineered skin to increase its thickness, the cTES was placed at the air–liquid interface to promote keratinocyte maturation and epidermal cell differentiation (Figure 1B).

### 2.2. Subcutaneous Injection of HeLa Cells Leads to Variable Rates of Tumor Development

**Figure 2:**
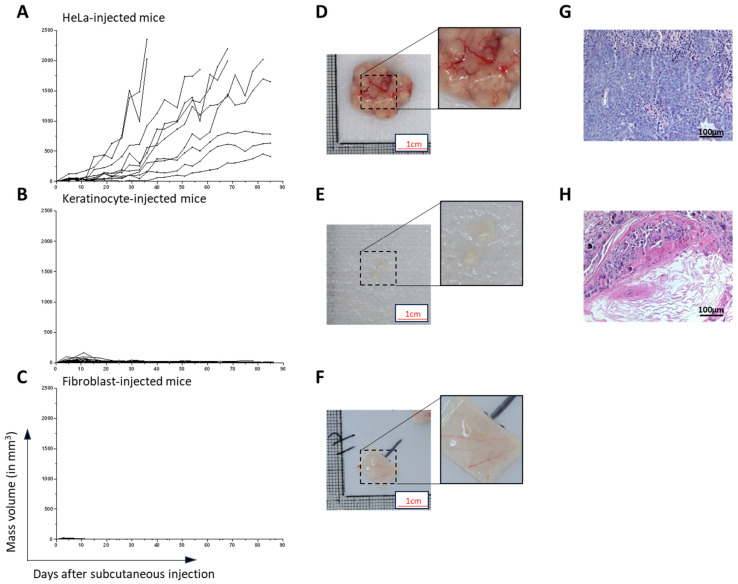
Evaluation of Mass Development After Injection In Vivo. (**A**–**C**) Evaluation of mass growth over time following subcutaneous injection of 10 million HeLa cells (**A**), healthy keratinocytes (**B**) or healthy fibroblasts (**C**). (**D**,**E**) Representative pictures of the tumor formed in HeLa cell-injected mice (**D**) or residual mass formed by keratinocyte-injected mice (**E**) after 3 months. (**F**) Representative injection site without any visible mass in fibroblast-injected mice after 3 months. (**G**,**H**) Hematoxylin and eosin staining of the mass formed in HeLa- and keratinocyte-injected mice.

The tumorigenic potential of HeLa cells was assessed using the subcutaneous injection method. After the injection, tumor growth over time was recorded by measuring the length, width and height of the bulge. Its volume was calculated using the formula for an ellipsoid. The volume measurement was found to be an accurate approximation of the tumor weight as assessed using correlation analysis of the estimated weight based on the volume and actual weight of the mass upon retrieval (Figure S1). All 10 mice injected with HeLa cells developed a tumor (Figure 2A,D). However, the growth rate was highly variable despite using the same number of HeLa cells from the same culture batch injected in mice of similar age (6 weeks). Six mice were euthanized before the 3-month mark (days 38, 58, 71 and 85) due to the mice reaching endpoints which were set to prevent animal distress, pain or interference with normal bodily functions. At sacrifice, tumors had a mass of 1.565 g (standard deviation (SD) = 0.75) (Figure S1). A histopathological analysis revealed the presence of a tumor formed by the injected HeLa cells in all replicates (Figure 2G). In contrast, the mice injected with 10 million healthy fibroblasts or healthy keratinocytes did not develop a tumor mass, indicating a lack of tumorigenic potential (Figure 2B,C). A small bulge was observed upon injection but shrunk over time for both fibroblasts and keratinocytes. In keratinocyte-injected mice, cells formed a keratinizing cyst (Figure 2H), whereas in fibroblast-injected mice no bulge could be found (Figure 2C,F).

### 2.3. In Vitro Development of HeLa Spheroids

**Figure 3:**
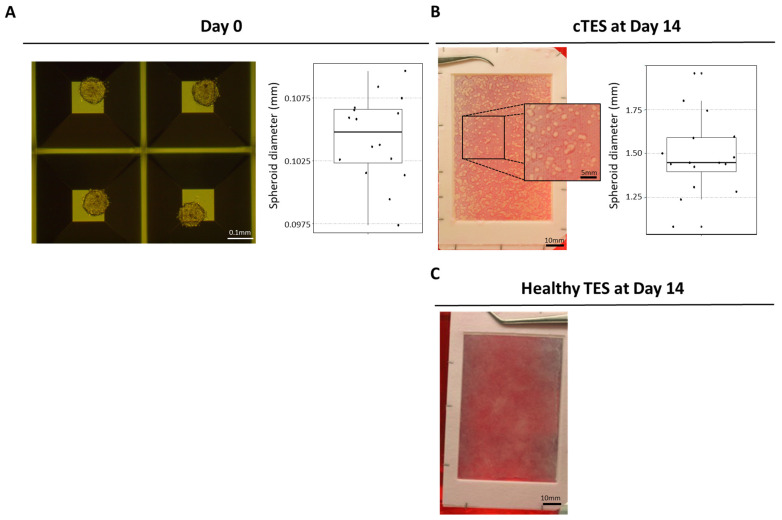
HeLa Spheroid Size Analysis. (**A**) HeLa spheroids’ appearance and diameter before seeding on dermal sheets (day 0); (**B**) macroscopic appearance of cTES cultured 14 days at the air–liquid interface and spheroid diameter before grafting; (**C**) macroscopic appearance of TES produced with healthy cells.

HeLa cells were used to produce spheroids. After two days of culture in Aggrewell plates, HeLa cells spontaneously arranged in spheroids (Figure 3A) of around 0.104 mm (SD = 0.003) in diameter. Once seeded at 25 spheroids per cm^2^ on a reconstructed dermal sheet produced with healthy fibroblasts, the HeLa spheroids adhered and proliferated. The macroscopical appearance of cTESs (Figure 3B) with round yellow spots formed by the HeLa spheroids was drastically different from TESs produced with healthy fibroblasts and keratinocytes (Figure 3C). During the period of cTES culture at the air–liquid interface, which serves to promote the maturation of the epidermis, the spheroids continued to grow to reach a diameter of 1.48 mm (SD = 0.22) at the time of grafting (Figure 3B).

### 2.4. cTES Grafting Allows Rapid Growth of Cancer Cells

**Figure 4:**
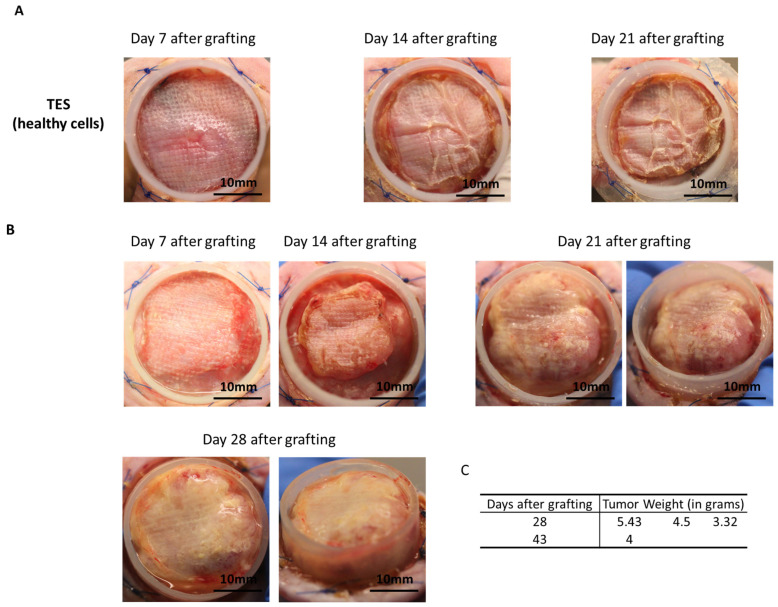
Appearance of TES or cTES and Tumor Weight After Grafting. (**A**) Macroscopic appearance of healthy TES at day 7, day 14 and day 21 after grafting; (**B**) macroscopic appearance of cTES at day 7, day 14, day 21 and day 28 after grafting; (**C**) tumor weight of cTES (in grams) at sacrifice for the four grafted mice (day 28 or day 43 after grafting).

**Figure 5:**
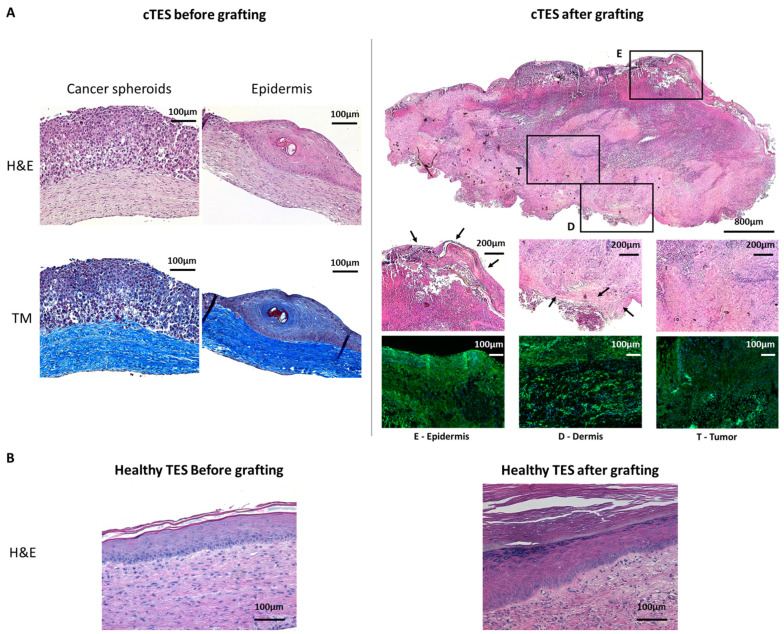
Histological Analysis Before and After Grafting. (**A**) Hematoxylin and eosin (H&E) and Masson trichrome (MT) staining of cTES before grafting. Hematoxylin and eosin staining (H&E) and immunofluorescence staining against HLA-ABC (green) of the cTES graft site 28 days after grafting. Arrows point to the epidermal remnants in cTES. Cell nuclei were stained with Hoechst (blue). (**B**) Hematoxylin and eosin staining (H&E) of healthy TES before and after grafting. E: epidermis; D: dermis; T: tumor.

To assess whether cancer cells could result in tumor formation within skin substitutes after grafting, cTESs were grafted on athymic mice and compared to TES produced from healthy cells. Seven days after grafting, cTESs were macroscopically similar to skin constructs produced without HeLa spheroids (Figure 4A,B). However, 14 days after grafting, cTESs were noticeably thicker than TESs. The cTESs continued to thicken and eventually filled the inside of the Fusenig chamber (5 cm^3^) after 28 to 43 days, at which point the mice had to be euthanized to prevent distress. Tumors developed rapidly, with a mean weight of 4.41 g (SD = 1.05) 28 days after grafting (Figure 4C). HeLa spheroids took over the skin substitutes and only remnants of cornified epidermis were observed on histological staining (Figure 5A). In contrast, no tumor developed on mice grafted with TES. A histological analysis confirmed that both TES layers became successfully integrated and survived after grafting onto athymic mice (Figure 5B). The dermis and the epidermis with the expected layers (basal, spinosum, granulosum and corneum) were present. 

A histological analysis indicated that before grafting, cancer spheroids formed unorganized clumps of HeLa cells that did not digest the underlying dermis (Figure 5A). The surrounding keratinocytes were able to differentiate into a mature epidermis. However, some cysts were present with terminally differentiated cells found within the epidermis of cTES (Figure 5A) in contrast to the healthy TES in which terminally differentiated cells were located in the uppermost epidermal layers (Figure 5B) as in native skin. After grafting, the spheroids rapidly developed into a large mass (Figure 5A) and the epidermal layer terminally differentiated into a corneal layer. The human origin of the cells was confirmed using human leukocyte antigen A, B, C staining (HLA-ABC).

### 2.5. Absence of Metastasis in Both Subcutaneous Injection and cTES Grafting Assays

**Figure 6:**
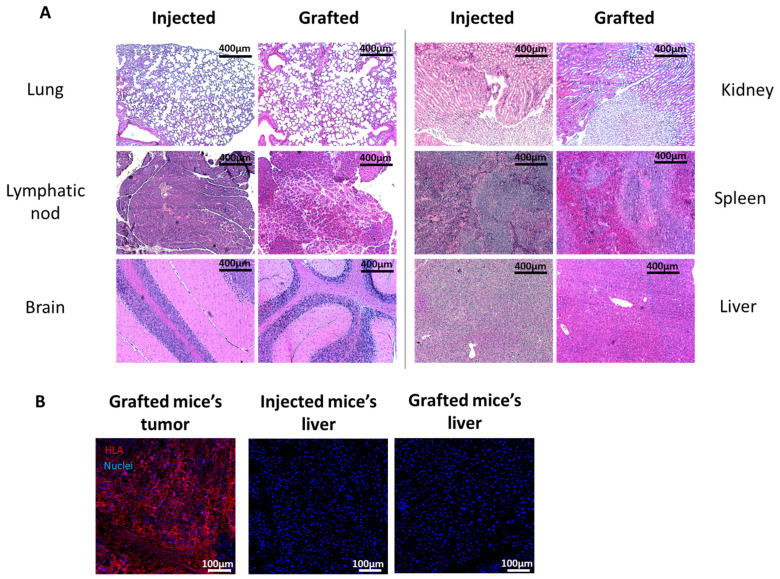
Histology for Metastasis Analysis of Mice Injected with HeLa Cells or Grafted with cTES. (**A**) Hematoxylin and eosin staining of organs (lung, lymph node, brain, kidney, spleen and liver) from mice grafted with cTES or injected with HeLa cells. (**B**) HLA-ABC (red) immunofluorescent staining of organs from mice grafted with cTES or injected with HeLa cells. Nuclei were stained with Hoechst (blue). The primary tumor of mice grafted with cTES was used as a positive control for HLA-ABC staining.

A histopathological analysis of organs (lung, lymphatic nodes, brain, kidney, spleen and liver) from mice that were injected with HeLa cells or grafted with cTES revealed no sign of a metastasis (Figure 6A). Moreover, there were no apparent human cells within the lumen of the blood vessels (lymphatics, blood) within and at the vicinity of the mass. Tumor formation was observed only at the injection or grafted site. HLA-ABC immunofluorescence staining revealed no human cells in organs (lung, brain, kidney, spleen and liver) of mice that were injected or grafted with cTES, confirming the absence of a metastasis (Figure 6B).

## 3. Discussion

HeLa cells were chosen to evaluate a new assay for tumorigenicity. This human cell line was selected for its known tumorigenic potential. Herein, we provided a proof of concept indicating that when cancer cells are present in tissue-engineered skin substitutes, they rapidly develop in vitro and in vivo after being grafted onto athymic mice. Based on the number of spheroids used in cTES production, 0.125 million cells cultured for 23 days in vitro (2 days in Aggrewell, 7 days on the dermal sheet and 14 days at the air–liquid interface) was sufficient to produce large tumors (4.41 g, SD = 1.05) 28 days after grafting. In contrast, the injection of 10 million cells required up to 3 months to cause tumor development. Even after 3 months, 3 of the 10 mice developed relatively small tumors compared to the ones formed 1 month after grafting cTES. For comparison, the injection method led to the development of masses with a mean volume of 362 mm^3^, which represents a mass of 0.362 g, (SD = 0.811 g), 31 days after injection, and is 12 times lighter. The coefficient variation of the standard deviation to the mean was 223.6% in injected mice 31 days after injection, compared to 23.9% in cTESs 28 days after grafting, indicating that tumor growth was more similar in the group of mice grafted with cTES. We cannot exclude that in athymic mice there is a difference in the capacity of immune cells to react to the presence of tumor cells in cTES compared to subcutaneously injected HeLa cells. However, we did not observe a difference in immune infiltration histologically. Moreover, we have shown previously that allogeneic epidermal cells within TES are rejected when transplanted on immune competent mice [20]. Therefore, we believe that a difference in the immune system’s attack due to the method of transplantation (injection vs. tissue grafting) is unlikely to be the explanation for the difference in tumor growth. Together, this indicates that using cTESs could be a sensitive and reproducible assay with which to determine the tumorigenic potential of epithelial cells. The more rapid growth of the tumor within the cTES after grafting compared to the injection assay could be due to the three-dimensional organization of cells within spheroids and in the cTES before grafting. The cell adhesion and polarity within cTESs may protect them from the cell death that could occur after cell injection. The more compact organization, as well as the surrounding normal cell environment, perhaps favors the biological signals required for survival and proliferation. A rapid vascularization of the cTES after grafting could also explain the difference in growth rates. Another advantage of grafting cTES is that the graft is easily identifiable. It could help recuperate the graft before the mass becomes too big. Small tumors could be more easily identified. The waiting time could then be decreased, which could prevent endpoints being reached. Therefore, this model could have ethical advantages for the use of animals. 

HeLa cells were chosen in this study due to their epithelial origin, because they are widely used as a positive control in tumor formation experiments, and they are well characterized [21]. However, future experiments with multiple tumor cell populations, particularly from skin cancer, such as squamous cell carcinoma and melanoma, will be required to draw definitive conclusions. The cTES represents a modeling method that may be more faithful to human skin cancer than in the injection method, mimicking the normal microenvironment. In turn, cTESs use both in vitro and in vivo could be a powerful tool for the investigation of new drugs in treating skin cancers. A combination of cTES and multicellular tumor spheroid may increase the fidelity of the model, with the presence of cancer-associated fibroblasts, for example, which have been shown to promote invasion [22,23,24,25,26]. Moreover, the sensitivity of this method could be assessed to determine how many cancer cells are necessary to generate a visible mass within 1 or 3 months, and whether this method is also more sensitive than the subcutaneous injection assay for the detection of the tumorigenic potential of a variety of other cancer cell populations. The grafting of skin substitutes on animals is routinely performed for various purposes, including investigations of the impact of specific modifications to production protocols, cell culture conditions, gene therapy effectiveness for genodermatoses [19], and more fundamental research and preclinical studies. Moreover, a large number of mice is required for the tumorigenic assay using the cell injection method, with 10 mice required for each cell type. For bilayered skin substitutes that contain at least two cell types, this means an injection of a positive control, as well as untreated fibroblasts and keratinocytes, for a total of 30 mice, and if fibroblasts and keratinocytes are treated, 20 more mice are injected. If future experiments show that skin cancer cells effectively grow well in grafted skin substitutes, the use of TES grafting to evaluate the growth of cancer cells could result in a new model for the evaluation of the tumorigenic potential of skin substitutes. Then, mice grafted with TES to assess the functionality and long-term survival of the skin substitute could also be used to provide results for the tumorigenic assay. This would reduce the number of mice required for pre-clinical studies, since only the addition of the cTES control would be necessary instead of using additional mice for the injection of each normal cell type. In addition, if this new assay is revealed to be sensitive enough for various types of cancer, it could also reduce the time and cost of tumorigenic assays.

It is possible that our model is more sensitive for tumors of epithelial origin, but it is not restricted to skin cancer models, since we used HeLa cells originating from a cervix tumor. Some cancer cells may not react well or be able to grow in our culture conditions, for example, at the air–liquid interface, a condition that is applied to the cTES both in vitro and in vivo. We believe that this method will be useful for skin cancer because TES mimics the cellular environment of the cancer (presence of a dermis, vascularization and keratinocytes). However, for other types of cancer, more experiments will be necessary to determine whether tumor growth is limited by the presence of skin cells or the air exposure, which may not be a truthful representation of the cancer’s environment.

## 4. Materials and Methods

### 4.1. Ethical Considerations

These experiments were conducted in agreement with the Declaration of Helsinki and our institution’s guidelines. This study was approved by our institution’s protection of human participants (Comité d’éthique de la recherche du CHU de Québec—Université Laval: No. 2012-1248; 28 April 2012 and approved yearly since 2012) and animal care (Comité de protection des animaux de l’Université Laval: no. CHU-22-1068; 22 November 2022) committees. Written informed consent was obtained for the use of retrieved skin tissues for research and educational purposes.

### 4.2. Cell Culture

Human keratinocytes and fibroblasts were isolated from the foreskin of a healthy individual (14 days old) as previously described [27]. Primary fibroblasts and HeLa cells (ATCC CCL-2) were cultured in fibroblast medium (Dulbecco-Vogt modified Eagle medium (Gibco™, Waltham, MA, USA) supplemented with 10% Avantor Seradigm FB Essence serum (Avantor^®^, Radnor, PA, USA), 100 U/mL penicillin (Sigma-Aldrich, St. Louis, MO, USA) and 25 μg/mL gentamicin (Gemini Bio, West Sacramento, CA, USA)). Human keratinocytes were cultured on a feeder layer composed of irradiated human fibroblasts cultured in keratinocyte medium (Dulbecco-Vogt modified Eagle medium (Gibco™): Ham’s F12 (Gibco™), ratio 3:1, supplemented with 24.25 μg/mL adenine (Sigma-Aldrich), 5 μg/mL insulin (Sigma-Aldrich), 0.4 μg/mL hydrocortisone (Galenova, Saint-Hyacinthe, QC, Canada), 0.212 μg/mL isoproterenol hydrochloride (Sigma-Aldrich), 5% bovine HyClone FetalClone II serum (GE Healthcare, Chicago, IL, USA), 10 ng/mL human epidermal growth factor (Austral Biologicals, San Ramon, CA, USA), 100 U/mL penicillin (Sigma-Aldrich) and 25 μg/mL gentamicin (Gemini Bio)).

### 4.3. Spheroid Formation

HeLa cells were seeded in AggreWell plate (AggreWell 800 24-well plate, StemCell Technologies, Vancouver, BC, Canada) at a density of 1000 cells/microwell following manufacturer’s recommendations. Briefly, AggreWell plates were washed with anti-adherence rinsing solution (500 µL/well) and spun for 5 min at 1300× *g*. Wells were washed twice with 2 mL fibroblast medium. HeLa cells cultured in T75 cm^2^ flasks (Corning, Corning, NY, USA) were detached near confluency using trypsin (0.05%)—EDTA (0.01%). Suspended cells were seeded in AggreWell plates and spun for 3 min at 100× *g*. After 2 days, spheroids were detached from the microwell by gently pipetting media on the microwells.

### 4.4. TES and cTES Production

Tissue-engineered skin substitutes (TESs) were produced as previously described [27]. Briefly, fibroblasts were seeded at 4 × 10^3^ cells/cm^2^ onto an 85 cm^2^ rectangular culture-treated dish. A hollowed-out anchoring paper (grade 237 filter paper, Ahlstrom) was placed at the bottom of the dish to be able to manipulate the reconstructed tissue. Fibroblasts were cultured for 28 days in fibroblast medium supplemented with 50 μg/mL of ascorbic acid. Keratinocytes were then seeded at 100,000 cells/cm^2^ onto the dermal sheets. Dermal sheets with keratinocytes were then cultured in keratinocyte medium supplemented with 50 μg/mL of ascorbic acid for 4 days before stacking this sheet onto 2 other dermal sheets to increase dermal thickness. TESs were then cultured at the air–liquid interface for 14 days in keratinocyte medium supplemented with 50 μg/mL of ascorbic acid without EGF.

For cancerous tissue-engineered skin substitutes (cTESs) fibroblasts were seeded at 4 × 10^3^ cells/cm^2^ onto an 85 cm^2^ rectangular culture-treated dish containing a hollowed-out anchoring paper (grade 237 filter paper, Ahlstrom, Kaukauna, WI, USA). Fibroblasts were cultured for 35 days in fibroblast medium supplemented with 50 μg/mL ascorbic acid. Spheroids were gently removed from the AggreWell plate 2 days after seeding and counted using a hemocytometer. Spheroids were seeded at a density of 25 spheroids/cm^2^ and left to adhere for 3 days. On day 38, keratinocytes were added at 100,000 cells/cm^2^ onto the dermal sheets previously seeded with spheroids. Dermal sheets with spheroid and keratinocytes were then cultured in keratinocyte medium supplemented with 50 μg/mL ascorbic acid for 4 days before stacking this sheet onto 2 other dermal sheets. cTESs were then cultured at the air–liquid interface for 14 days in keratinocyte medium supplemented with 50 μg/mL ascorbic acid without EGF.

### 4.5. Subcutaneous Injection of Nude Mice

Cultured cells were detached using trypsin (0.05%)—EDTA (0.01%) after massive amplification in multi-layer flasks (875 cm^2^ Cell Culture Multi-Flask, Corning, Corning, NY, USA). Suspended cells were counted using a Coulter Cell Counter and viability was assessed using trypan blue. Cells were centrifuged for 10 min at 300× *g* and resuspended at a density of 1 × 10^7^ cells per 200 µL in serum-free fibroblast culture media for HeLa cells and fibroblasts, or in serum-free keratinocyte medium for keratinocytes. Mice were anesthetized using isoflurane inhalation and 200 µL of the cell suspension was injected subcutaneously, within an hour after cell detachment from the culture flasks. Permanent skin tattoos were done prior to cell injection to track the injection site. At least 10 mice were injected in each group.

### 4.6. Tumor Size Measurements

For each mouse, the mass formed after the subcutaneous injection was measured twice a week. Using a caliper, the length, width and height of the mass was measured. The volume was estimated using the formula of an ellipsoid:

$$\frac{Length\;\left(in\;mm\right)\;\times \;Width\;\left(in\;mm\right)\;\times \;Height\;\left(in\;mm\right)\;\times \;\pi }{6}$$

### 4.7. Skin Substitute Grafting onto Nude Mice

Each cancerous skin substitute was grafted onto the back of a nude athymic mouse (Crl:CD1-Foxn1^n^; Charles River Laboratories, Laval, QC, Canada) within a Fusenig chamber, allowing to encase the skin substitute as previously described [27]. Briefly, a 2.5 × 2.5 cm square was cut out of the skin substitute. A sterile gauze (AdapticTM, Acelity, Mississauga, ON, Canada) was placed on the epidermis of the substitute and secured with ligating clips (Ligaclip, Ethicon, Raritan, NJ, USA). The cTES was prepared 24 h prior to grafting and stored on a DMEM-agar gel (DMEM medium with 0.75% agarose). Ligaclips were removed with scissors before grafting. Each cTES was placed on the inside of the Fusenig chamber and secured using a bolster dressing. The bolster dressing was removed after 7 days. After 28 to 43 days, each mouse was euthanized, and the graft was removed for analysis. 

### 4.8. Histological and Immunofluorescence Analysis

Injected sites, TES and cTES biopsies before and after grafting and organs after injection or grafting were fixed in 3.7% formaldehyde (pH = 7) and embedded in paraffin. Five micrometer sections were stained with hematoxylin and eosin or Masson’s Trichrome. Biopsies were also embedded in Tissue-Tek optimal cutting temperature Compound (Sakura, Finetek, Torrance, CA, USA), frozen in liquid nitrogen and stored at −80 °C. Five micrometers cryosections were permeabilized with acetone for 10 min at −20 °C. Sections were washed with phosphate-buffered saline (PBS) and incubated with a blocking buffer (2% *w/v* bovine serum albumin (BSA) in PBS) (30 min). Antibodies were diluted in blocking buffer. For HLA staining of injection and grafted sites, sections were incubated with an anti HLA-ABC 1:25 (Biolegend, San Diego, CA, USA) for 60 min, washed in PBS and incubated with Alexa 488-conjugated goat anti-mouse antibody at a working dilution of 1:800 for 45 min (Invitrogen, Waltham, MA, USA). For HLA staining of mice organs, sections were incubated with PE/Cyanine5-conjugated mouse anti-HLA-ABC antibody (Biolegend, San Diego, CA, USA) for 60 min. Cell nuclei were stained with Hoechst (Sigma-Aldrich, Saint-Louis, MO, USA).

## 5. Conclusions

In conclusion, the clinical significance of this model is in the domain of cell- or tissue-based therapies and regenerative medicine. New tissue-engineered substitutes have to be tested for their tumorigenic potential before they can be used in clinical trials. More complex tissue substitutes comprising a larger number of cell types are being produced with the progress in stem cells and tissue engineering. The use of this new model could be an alternative to reduce the number of animals, the cost and the time necessary for quality control testing, since it remains sensitive to tumor growth even if several cell types are present. It will then be useful in the elaboration of new treatments arising from tissue engineering. 

In the future, another application of this model could be anti-cancer drug testing. However, further development is required. Testing with skin cancer cells and exposure to known anti-cancer drugs should be performed before drawing general conclusions.

## Data Availability

The data presented in this study are available upon request to the corresponding author.

## Supplementary Materials

The following supporting information can be downloaded at: https://www.mdpi.com/article/10.3390/ijms25031513/s1.
