# Supplementary material for: Cancer Spheroids Embedded in Tissue-Engineered Skin Substitutes: A New Method to Study Tumorigenicity In Vivo
Source: Int J Mol Sci. 2024 Jan 26;25(3):1513. doi: 10.3390/ijms25031513 (PMC10855415; doi:10.3390/ijms25031513)
Supplement: Supplementary file 1 [file ijms-25-01513-s001.zip › ijms-2823094-supplementary.pdf]

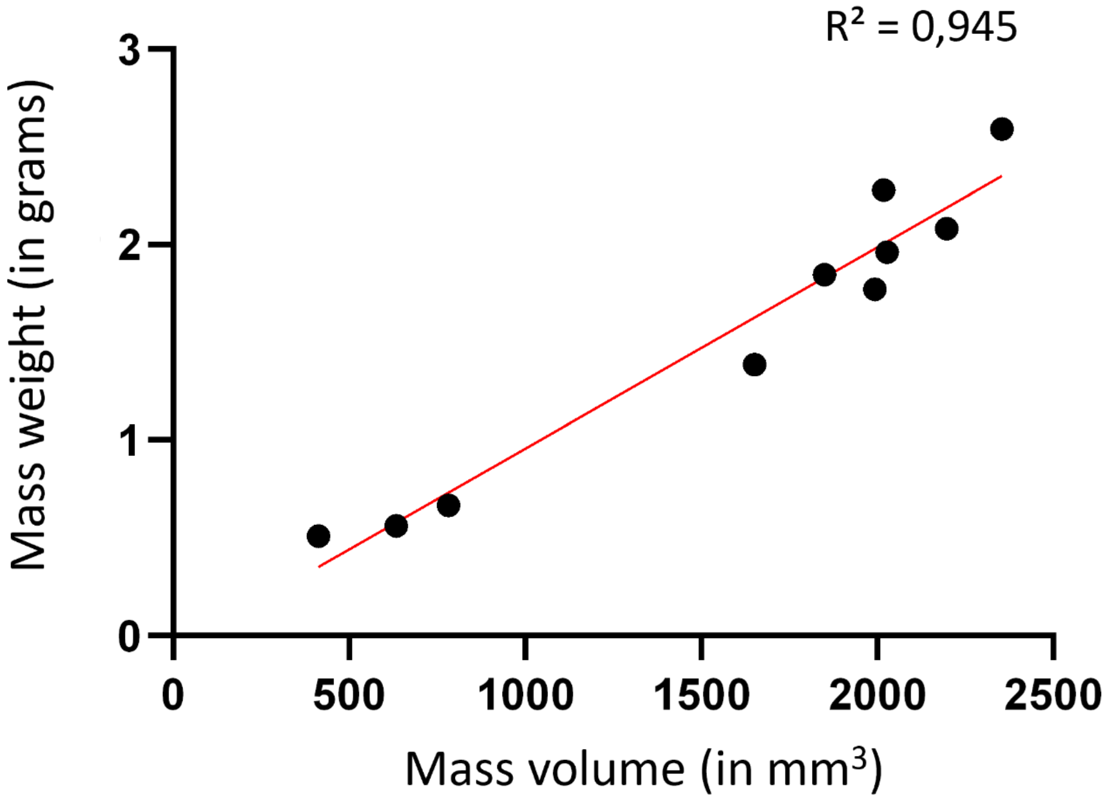

Figure S1. Correlation analysis of weight versus volume measurements. Volumes were estimated using length, width and height of the mass and the formula for an ellipsoid (length x width x height x pi/6). Tumors were weighted after retrieval. Spearman's correlation analysis showed significant correlation (r=0,9515, P-value < 0,001) and simple linear regression showed a R^2 of 0,9451. Linear curve is shown in red.
